# To Sweep and Dust

**Published:** 1881-11

**Authors:** 


### TO SWEEP AND DUST.

SWEEPING and dusting is, in the opinion of the Alliance, an art, and has a right method which is as follows :

Are there closets opening into the room to be swept ? Arrange the shelves, drawers or clothing preparatory to sweeping-day ; then let these be first to be swept.

Cover the bed with soiled sheets, as also all heavy articles that cannot be removed ; first, however, having carefully dusted and brushed them.

Remove all the furniture that can easily be set in the hall or adjoining room, having first dusted it ; then, taking a step-ladder, begin to sweep or brush or wipe the cornice and picture-cords and pictures.

Draw the shades to the top of the window, or, if they are inside blinds, dust them carefully. Open the windows.

All the dust left in the room now is in the carpet or air, and the current of the windows will soon settle it.

Now begin to sweep, not toward a door or corner, but from the outer edges of the room toward the centre, where the dust will be taken up with a small brush or dust-pan.

Go over the room once more, this time with a dampened broom; that removes the last bit of dust, and gives the carpet a new, bright appearance.

Replace the articles of furniture as soon as the air is entirely free from dust, uncover the rest, and the room is new and clean.

All this seems an easy thing to do, but there is not one in a hundred will follow out the detail.

Some will sweep the dust into the hall or from one room to another, and then wonder why their house is so soon dusty again.

Others forget cornice and pictures, and thus leave a seed for future annoyance ; while a third class will do all but using the damp broom, which is as the finishing touches to a picture.

---

ORIGIN OF "HURRAH."—Thousands of people have shouted "hurrah !" "many a time and oft," but comparatively few know its derivation and primary meaning. It originated among the Eastern nations, where it was used as a war-cry, from the belief that every man who died in battle for his country went to Heaven. It is derived from the Sclavonic word Hurraj, which means "to paradise."

